# Software Tools for 2D Cell Segmentation

**DOI:** 10.3390/cells13040352

**Published:** 2024-02-17

**Authors:** Ping Liu, Jun Li, Jiaxing Chang, Pinli Hu, Yue Sun, Yanan Jiang, Fan Zhang, Haojing Shao

**Affiliations:** 1 College of Computer Science and Technology (College of Data Science), Taiyuan University of Technology, Jinzhong 030600, China; liuping01@tyut.edu.cn (P.L.); lijun0418@link.tyut.edu.cn (J.L.); changjiaxing@caas.cn (J.C.); 2 Shenzhen Branch, Guangdong Laboratory of Lingnan Modern Agriculture, Genome Analysis Laboratory of the Ministry of Agriculture and Rural Affairs, Agricultural Genomics Institute at Shenzhen, Chinese Academy of Agricultural Sciences, No 7, Pengfei Road, Dapeng District, Shenzhen 518120, China; hupinli@caas.cn (P.H.); sunyue@caas.cn (Y.S.); jiangyanan@caas.cn (Y.J.); griffanzhang2013@gmail.com (F.Z.)

**Keywords:** cell segmentation, image processing, 2D cell, performance

## Abstract

Cell segmentation is an important task in the field of image processing, widely used in the life sciences and medical fields. Traditional methods are mainly based on pixel intensity and spatial relationships, but have limitations. In recent years, machine learning and deep learning methods have been widely used, providing more-accurate and efficient solutions for cell segmentation. The effort to develop efficient and accurate segmentation software tools has been one of the major focal points in the field of cell segmentation for years. However, each software tool has unique characteristics and adaptations, and no universal cell-segmentation software can achieve perfect results. In this review, we used three publicly available datasets containing multiple 2D cell-imaging modalities. Common segmentation metrics were used to evaluate the performance of eight segmentation tools to compare their generality and, thus, find the best-performing tool.

## 1. Introduction

Cell segmentation is an important step for imaging studies and is widely used in the life sciences, bioinformatics, and biomedical fields such as oncology, immunology, and histopathology, including the emerging field of spatial transcriptomics. Scientists apply cell segmentation and prior knowledge of cell-type-specific gene expression to analyze the morphology and location of individual cells, obtain single-cell gene counts, and detect fine intracellular variations [1,2,3].

Differences in biological tissue, intercellular heterogeneity, and high cell densities need to be resolved before analyzing cell imaging data. Further, differences in illumination gradients, imaging modalities, and imaging parameters have to be accounted for while considering segmentation solutions [4,5]. In addition, microscopic techniques for cell imaging have improved, resulting in higher resolution, broader visualization, and noninvasive cell images. Common microscopic techniques include bright field microscopy, fluorescent microscopy, confocal microscopy, and phase contrast microscopy [6,7,8,9]. The more-recent imaging methods, such as cell staining combined with immunohistochemistry (IHC), multiplex immunofluorescence (mIF) [10], and CO-Detection by IndEXing (CODEX) [11] can co-detect and co-locate multiple transcriptomes and proteins, resulting in the precise annotation of individual cell types and the resolution of biological functions. In general, cell images obtained using such techniques have different channel types. The main image channels include three-channel RGB, three-channel HSV, single-channel GRAY, and fluorescently labeled specific channels, which are more commonly used by researchers.

Most of the traditional segmentation methods are based on the intensity and spatial relationship of pixels, and the constraint model is found by manual optimization, requiring expertise in basic techniques including code adaptation [12]. Code adaptation is highly subjective, and its development has reached a bottleneck. For example, the threshold only considers the grayscale information of the image and is sensitive to noise, which can easily cause uneven segmentation results; the region algorithm has low adaptability and performs poorly when used alone; the watershed algorithm is susceptible to over-segmentation due to noise; the graph theory algorithm is complex, computationally intensive, and not easy to operate. Therefore, using a sequential combination of these algorithms can effectively avoid these deficiencies and further separate touching or overlapping cells [13]. In addition, threshold processing and watershed algorithm are often used as preprocessing or postprocessing methods for Machine Learning (ML) and Deep Learning (DL).

ML and DL have similar workflows: the selection of the training data, data processing, model training, and model evaluation. These steps in the workflow may be iterative until the model is appropriate and accurate. HK-means [14], Random Forests [15], and EM [16] are trainable ML methods that include part of the knowledge in the segmentation process and improve the Accuracy of the segmentation. DL algorithms are better suited to addressing the challenges of cell segmentation, including multiple object morphologies and imaging techniques. DL network structures provide a generalized framework that can be applied to various tasks in different domains. They learn from data, adapt to different problem settings, and leverage the capabilities of pre-trained models, making them a convenient and effective strategy in many research and application fields. Further, manual tuning is not needed; however, retraining with annotated data is required [12].

The core algorithms in 2D segmentation tools are gradually shifting to more-complex deep learning networks. At the beginning, the earlier 2D segmentation tools CellProfiler [17] and Icy [14] used built-in traditional segmentation algorithms, such as watershed algorithms. Later, the classic U-net [18] structural deep learning model was wildly used and improved. StarDist added a polygon distance output layer [19,20]. Cellpose replaced the standard building blocks with residual blocks [21,22]. Notably, many 2D segmentation tools keep adoptingthe best segmentation models and update their software. For instance, CellProfiler and Icy update their deep learning model plugins (ClassifyPixels-U-net, DoGNet, etc.) for cell segmentation.

For this study, we used three publicly accessible datasets with annotations from several cell-imaging modalities to compare the generality of the tools.

## 2. Selected Software for Performance Comparison

As shown in Table 1, the selection of the segmentation software for the quantitative comparison was determined by three factors: (1) the ability to analyze cell images from various cell types; (2) those most-commonly used by scientists, especially for biological cell image segmentation; and (3) the feasibility of the installation, training, or use. The summary of the user-friendliness of the software is given in Table 2.

### 2.1. CellProfiler

CellProfiler (version 4.2.6) is a modular, high-throughput, and open-source software that provides a user-friendly interface, supports automatic cell identification and segmentation, and enables various feature extractions and data analyses, such as morphological measurements and cell counting. CellProfiler supports a variety of cell segmentation algorithms such as threshold algorithms, morphology algorithms, watershed algorithms, etc. It is convenient for users to choose the most-suitable algorithm or combination of algorithms according to different needs and adjust the parameters to improve the segmentation Accuracy and reduce errors. The software also supports segmentation using mainstream DL models via plugins. Scientists run CellProfiler headless or with a pipeline that supports batch processing to analyze multiple image datasets simultaneously and improve the Accuracy and consistency of large-scale data analyses. In general, a segmentation pipeline consists of four steps. First, it applies morphological operations to remove image noise. Second, it utilizes the gradient intensity of the image to obtain edge information. Third, the IdentifyPrimaryObjects module performs binarization to automatically detect and segment cells in the image and label them and further removes signal noise or fills gaps using certain morphological operations. Finally, it converts the cell masks to uint16 TIFF format images for preservation. The software, user manuals, video tutorials, and pipeline examples are available at: https://cellprofiler.org/ (accessed on 8 February 2024).

### 2.2. Icy

Icy (version 2.4.3.0) is a free, user-friendly, open-source image-analysis software suitable for high-throughput cells. It provides advanced analysis features: cell localization, segmentation, tracking, morphological measurements, etc. Among the plugins or modules for segmentation are Thresholding, Active Contour Line, Parametric Snake, Potts Segmentation, Texture Segmentation, Watershed3D, Spot Detector, and HK-Means [31]. Icy provides a Graphical User Interface (GUI) based on visual programming that allows users to select protocols from existing site libraries or manually configure their workflows for subsequent batch reuse. Java-based and Python-based scripts may be used instead of the plugin modules. For this study, we used a protocol and selected the commonly used HK-Means segmentation, which carries out N-class threshold processing based on the K-Means classification of the image histogram. The threshold processing uses a method based on similarity measurement to assign different brightness values to the cell images and their backgrounds. The object was then extracted from the bottom up using a user-defined minimum and maximum object sizes (pixels) to eliminate groups that are too small or too large. Finally, Gaussian pre-filtering was applied to improve the segmentation of noisy images. Icy is developed in Java. Its source code, standalone versions for Windows and Mac, documentation, user manuals, and video tutorials are distributed at: https://icy.bioimageanalysis.org/ (accessed on 8 February 2024).

### 2.3. StarDist

StarDist (version 0.8.5) is a DL method for 2D and 3D object detection and segmentation, which can handle complex shapes and structures with high Accuracy and robustness and is applied to the life science and medical image processing. It uses a Convolutional Neural Network (CNN) to predict the distance of each pixel to the object boundary and determines the probability of it belonging to the target object. The program generates a star-shaped convex polygon or convex polyhedron to represent the detected object. StarDist supports the detection of irregularly shaped objects against complex backgrounds. An adaptive model of the dataset is obtained through the application of a training on the original image and corresponding annotated data. The model is then used to test new data for prediction and evaluation. For 2D data, the pre-trained model can be used directly. It has no GUI, but provides a Python-based toolkit that can be used as a plugin for common open-source platforms such as ImageJ/Fiji [32], CellProfiler, Icy, KNIME, QuPath, etc. The source code is available at: https://github.com/stardist/stardist (accessed on 8 February 2024).

### 2.4. DeepCell

DeepCell [23,24,25,26] (version 0.12.9) is an open-source Python package for automated analysis and segmentation of high-throughput biological images. The software uses Deep Convolutional Neural Networks (DCNNs) to perform cell segmentation on various types of microscopy images and predict cell types. It includes several functions, including cell segmentation, nucleus segmentation, cell tracking, and phenotype measurement. DeepCell supports high customizability and migratory learning, and users can train their Neural Network architectures using pre-trained models or the provided design rules for image normalization, data enhancement, hyperparameter tuning, and post-segmentation processing to improve Accuracy. DeepCell also provides visualization tools to help researchers better understand the output data. It has no GUI, but offers alternative ways to interface with it, including a web portal, ImageJ plugins, and command-line interfaces. Its documentation, user manuals, and tutorials are distributed at: https://deepcell.com/ (accessed on 8 February 2024).

### 2.5. Cellpose

Cellpose (version 2.2.3) is an open-source general-purpose cell-image-segmentation software that can be applied to a wide range of biological imaging modalities and is suitable for identifying microorganisms and plant and animal cell types. Cellpose uses a modified Neural Network architecture based on U-net. Unlike classical segmentation, which uses image grayscale values to create topological maps, Cellpose uses intermediate image representations to form smoother topological maps to better segment multiple types of non-clumped cells. In addition, Cellpose 2.0 provides a large dataset of trained “model zoos” containing several pre-trained models where users can choose the appropriate pre-trained model and evaluate their data. Pre-training scripts are also provided so that users can efficiently train custom models. Cellpose inputs the approximate average cell diameter in pixels or takes the model’s default values for single-channel images. Image resolution is limited to a maximum of 512 × 512 px, effectively reducing the computation time while bringing fewer segmentation errors. It is provided with a web portal, and a GUI can be found at https://www.cellpose.org/ (accessed on 8 February 2024).

### 2.6. Omnipose

Omnipose [27] (version 1.0.6) is a user-friendly cell segmentation tool based on DL, using the U-net architecture. Omnipose is based on Cellpose and comes with significant upgrades, such as a new postprocessing step that removes noise and fine-tunes segmentation contours. In addition, it handles images with uneven lighting or blurred backgrounds and is optimized for fast processing while maintaining high Accuracy. Omnipose has been used to segment bacteria in large bacterial image databases. Omnipose can robustly segment bacteria with different morphologies, outperforms Cellpose on bacterial phase contrast images, and provides convincing results even for highly eccentric or elongated cells. In addition, Omnipose can accept images of any size, improving training efficiency and allowing more cell detection. The output of Omnipose is an instance segmentation. By integrating Omnipose with other tools, scientists can determine the average pixel diameter of bacterial cells from real surface masks. Omnipose offers a GUI, command-line interface, and Jupyter notebook at https://omnipose.readthedocs.io (accessed on 8 February 2024).

### 2.7. Plantseg

Plantseg [28] (version 1.6.0) is a comprehensive software for the 2D or 3D cell segmentation of plant cells. Plantseg utilizes a pipeline that involves a CNN to predict cell contour boundaries and various graph-partitioning algorithms to segment cells based on the previous step. The software was designed to work with confocal microscope and light-sheet microscope images. Users have the option of using a pre-trained network or training a 2D U-net or 3D U-net [33] architecture for the first step. The segmentation algorithms available in Plantseg include GASP [34] (default), Mutex watershed, MultiCut, SimpleTK, and DtWatershed. Plantseg also uses distance-transformation-based watershed and region adjacency graphs to create superpixels for image segmentation. These techniques differ from traditional grayscale segmentation methods, such as extracting DAPI-stained cell nuclei. Plantseg is a free, open-source software that comes equipped with both a command-line and a GUI, making it accessible to a wide range of users. The source code is available at https://github.com/hci-unihd/plant-seg (accessed on 8 February 2024).

### 2.8. Ilastik

Ilastik [29,30] (version 1.4.0.post1) is a user-friendly, free, open-source software tool for interactive ML-based image analysis in a wide range of applications including neuroscience and cell biology. It enables researchers to perform complex image segmentation, classification, tracking, and counting of cells. Scientists can download the BioImage.IO library (DL models for the bioimaging community) in Ilastik. An interactive ML based on a Random Forest classifier with a GUI that does not require specific ML knowledge adjusts parameters with minimal manual annotation and human intervention, enabling users to implement their image analysis through a supervised ML workflow. Manual annotation is used to predict the class of each unannotated pixel and object, thus classifying pixels and objects and correcting them precisely at the locations where the classifier is wrong. Once the classifier has been trained, the new data can be processed in batch mode. We only successfully tested Ilastik on the Cellpose_cyto dataset without preprocessing operations. First, we used the pixel classification workflow to obtain initial semantic segmentations (two classes: cell and background). Then, we used boundary-based segmentation with the multicut workflow to produce the final cell instance segmentation. Ilastik runs on Windows, macOS, and Linux. User documentation and video tutorials are available at: https://www.ilastik.org/ (accessed on 8 February 2024).

## 3. Experimental Configuration and Analysis

### 3.1. Datasets and Preprocessing

**Figure 1:**
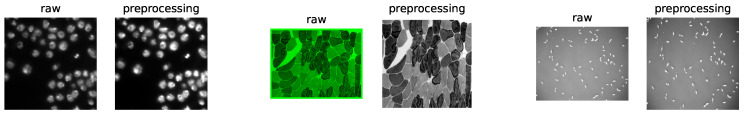
An example of raw images and preprocessing result of DSB2018 datasets (**left**), Cellpose_cyto datasets (**center**), and PhC-C2DL-PSC datasets (**right**).

As shown in Table 3, three public datasets were selected to evaluate eight cell-segmentation software tools. The first dataset was extrapolated from the 2018 Data Science Bowl [35]; the second was obtained from Cellpose [21]; the third was obtained from the Cell Tracking Challenge of ISBI [36]. The general H5 format image files are easy to process and effectively reduce memory consumption. However, since Icy does not support the input of H5 format files yet, we did not choose the H5 format. Instead, we took another commonly used TIFF image format for conversion. Because CellProfiler and DeepCell require the same-size input dataset for batch processing, we unified the different sizes of the dataset to 512 × 512 px and converted them to a single channel (y,x,c) for grayscale processing. Specifically, the input dimensions of Plantseg were z, y, and x, and those of DeepCell were b, y, x, and c, where “bzxyc” denotes the number of images, depth, height–width, and color channel, respectively. Finally, we normalized the minimum and maximum values of each image in the dataset after removing the upper and lower 1% extremes. We were, thus, able to minimize the impact of these differences in the image brightness and contrast and to ensure the stability and Accuracy of the software cell segmentation. For examples, see Figure 1.

### 3.2. Hardware Environment

The software was tested using two different hardware environments, as described below:

1. GPU:
  - System: Ubuntu 20.04.2 LTS;
  - GPU: NVIDIA GeForce RTX 3090 24 GB 2 GPUs;
  - RAM: 1 TB;
  - CPU: AMD EPYC 7H12 64-Core Processor;
  - CUDA: version 11.1.
2. CPU:
  - System: Windows 10;
  - CPU: Intel(R) Core(TM) i5-6200U CPU @ 2.30 GHz;
  - RAM: 2 × 4 GB 1867 MHz/s.

### 3.3. Segmentation Metrics

As in Table 1, we chose eight software tools to compare, selecting pre-trained models and the existing pipelines of the corresponding software, which use default parameters for cell image segmentation in both the models and pipelines. There are several metrics related to image segmentation in the field of computer imaging and ML/DL. In this paper, we chose some generic segmentation metrics [7]: Accuracy, Recall, Precision, and F1. As shown in Table 3, the DSB2018 dataset uses 50 images and the Cellpose_cyto dataset uses 68 images for the average metrics comparison.

$$\begin{array}{cc} \; & Accuracy=\frac{TP+TN}{TP+TN+FN+FP}\\ \; & Recall=\frac{TP}{TP+FN}\\ \; & Precision=\frac{TP}{TP+FP}\\ \; & F1=\frac{2*Recall*Precision}{Recall+Precision}=\frac{2*TP}{2*TP+TN+FN} \end{array}$$

## 4. Results

The default pre-trained model was selected for segmentation comparison. As shown in Table 4, Table 5, Table 6 and Table 7, we compared the segmentation results and conducted a quantitative comparison. Among these tables, N_true represents the number of cells in the Ground Truth (GT). N_pred represents the number of cells predicted by each software. Accuracy, Recall, Precision, and F1 are some segmentation metrics. F1-based Rank was used to compare the software performance based on the F1 score.

**Figure 2:**
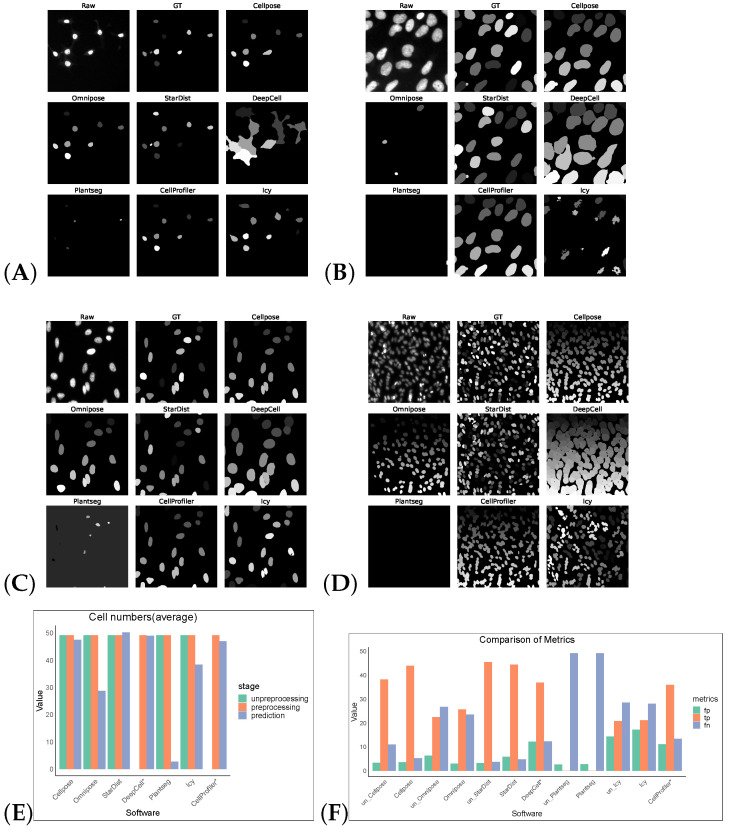
Some representative sections of the segmentation masks obtained after preprocessing the DSB2018 dataset as shown in (**A**–**D**). (**E**) shows the average cell numbers, and the suffix “*” indicates that the software had only successfully made segmentation predictions on preprocessed images. (**F**) shows FP/TP/FN values, and the prefix “un_” indicates that the software operates on non-preprocess images.

Without preprocessing, the top-three F1 scores of the software were for StarDist, Cellpose, and Omnipose. After the preprocessing operations, the F1 scores of the remaining five software tools, except for StarDist and Plantseg, improved, verifying the feasibility of the preprocessing operations. CellProfiler leverages the strengths of traditional algorithms, ranking first with an F1 score of 0.6394. StarDist was second with an F1 score of 0.5912, and Cellpose was third with an F1 score of 0.5763. The performance of the other software tools was comparatively low. The cell segmentation results using DeepCell expanded the boundary and, hence, it is more susceptible to noise, which makes the cells with obvious gaps appear adhesive and dense, resulting in over-segmentation. Icy is most affected by noise: the segmentation results contained holes and showed under-segmentation phenomena (Figure 2).

**Figure 3:**
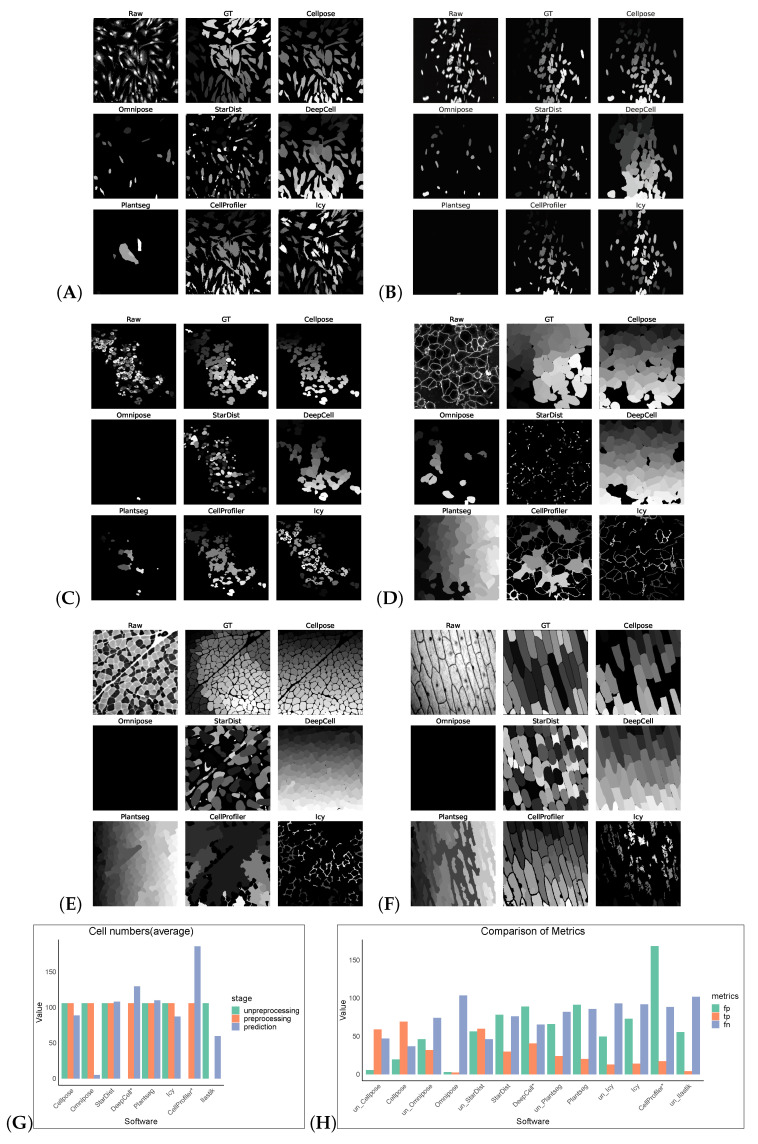
Some representative sections of the segmentation masks obtained after preprocessing the Cellpose_cyto dataset as shown in (**A**–**F**). (**G**) shows the cell numbers, and the suffix “*” indicates that the software had only successfully made segmentation predictions on preprocessed images. And (**H**) shows FP/TP/FN values, and the prefix “un_” indicates that the software operates on non-preprocess images.

Without preprocessing, the top-three F1 scores of the software were for Cellpose, Omnipose, and StarDist. The same preprocessing operation did not give good results on the Cellpose_cyto dataset, the input of was a three-channel image, which may also be a result of the diversity of its image types. After processing, Cellpose demonstrated the best performance with an F1 score of 0.6929 for the Cellpose_cyto dataset. DeepCell ranked second with an F1 score of 0.3847, followed by StarDist in third place with an F1 score of 0.3072. The performance of the other software tools was notably lower, with Precision, Recall, Accuracy, and F1 scores all falling behind the top-three performers. The poorer results of Omnipose compared to Cellpose, which was highly affected by preprocessing, may be related to its suitability for regular (round or oval) small cells and bacterial cells. Plantseg had the worst results for both datasets, which may be because it is a plant-cell-segmentation software, which is more adapted to the segmentation of tightly arranged cells. For neuron cells, apart from Omnipose, Plantseg and StarDist were among the software tools with poor segmentation results, which are more sensitive to differences in cell shape and more adapted to round or oval conventional cells. However, Icy and CellProfiler are more adaptable for such cells, with finer segmentation (Figure 3).

In addition, we selected two software tools with the best F1 scores (Cellpose and StarDist) to compare the training adaptability of their models with the default parameters and methods using two preprocessing datasets. After model training, the segmentation metrics of the two software tools significantly increased, as shown in Table 6. The F1 score of Cellpose rose from 0.5763 to 0.7026, surpassing the F1 score of StarDist (0.5912) on the DSB2018 dataset. The F1 score of StarDist rose from 0.3072 to 0.6739, lower than the F1 score of Cellpose (0.6929) on the Cellpose_cyto dataset.

**Figure 4:**
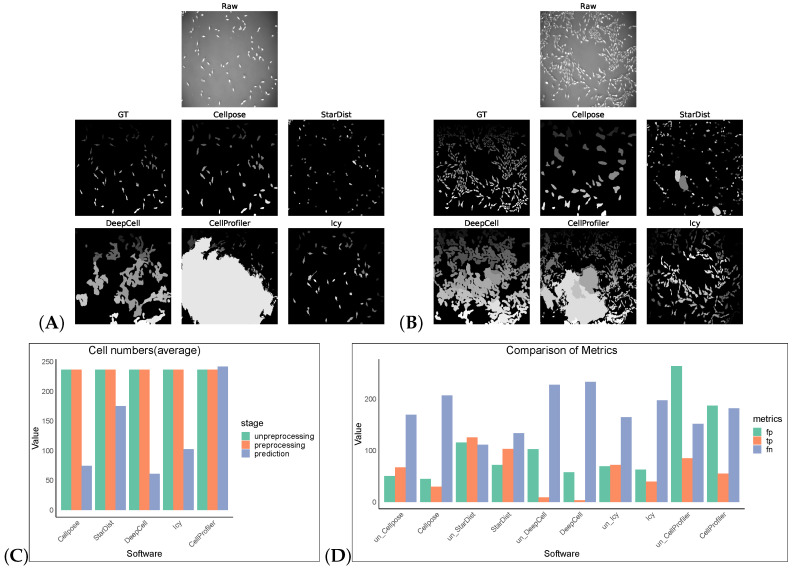
Some representative sections of the segmentation masks obtained after preprocessing the PhC-C2DL-PSC dataset as shown in (**A**,**B**). (**C**) shows the cell numbers, and (**D**) shows FP/TP/FN values, and the prefix “un_ ” indicates that the software operates on non-preprocess images.

We tested an additional 2D dataset (PhC-C2DL-PSC) for cell segmentation with and without preprocessing operations. This dataset was phase contrast images from pancreatic stem cells on a polystyrene substrate. We chose 300 images from its training dataset (folder 01) and the corresponding masks in folder 01_ERR_SEG. Since this dataset has a uniform size, DeepCell and CellProfiler could also perform segmentation without preprocessing. Omnipose and Plantseg among the selected software were ignored as there was no output in this dataset. The results are as shown in Table 7 and Figure 4. Without preprocessing, the top-three F1 scores of the software were StarDist, Cellpose, and Icy. The same preprocessing operation did not give good results on this dataset, except for StarDist.

Finally, we quantitatively compared the computer resources used to obtain the segmentation results for the DSB2018 and the Cellpose_cyto datasets with preprocessing for each of the selected segmentation software, as shown in Table 8. In a GPU hardware environment, Cellpose ran fastest on the DSB2018 dataset and second fastest to Omnipose on the Cellpose_cyto dataset. The maximum memory occupied during StarDist processing was the lowest on both datasets, except for Plantseg. Plantseg took up the least memory, but ran the slowest on both datasets.

## 5. Discussion

This study examined the performance of different cell-image-segmentation software tools on the DSB2018, the Cellpose_cyto, and the PhC-C2DL-PSC datasets. The DSB2018 dataset is generic, and the overall shape of the cells in the dataset is relatively uniform with differences in the gap densities and cell sizes. The Cellpose_cyto dataset comprises cells with various characteristics. The PhC-C2DL-PSC dataset is consistent on cellular features as it is a 2D time-lapse sequence of cell images. The final results showed performance differences across the software tools, with no one tool performing better than the others across all the measures and datasets evaluated. CellProfiler and StarDist performed well on the DSB2018 dataset, while Cellpose performed well on the Cellpose_cyto dataset, and Cellpose and StarDist performed well on the PhC-C2DL-PSC dataset. After systematic model training and learning, Cellpose and StarDist were trained on their respective datasets and showed similar performance on the two datasets. The segmentation result of Cellpose was slightly better than that of StarDist, indicating that Cellpose has better adaptability to different types of cells. Cellpose and StarDist use distance-transformed gradients to predict the final result, process faster during segmentation, and consume less memory resource, which can satisfy the researchers’ need for the batch processing of cells. Meanwhile, a more-complete process can be constructed using deep learning model extension plug-ins such as Cellpose and StarDist in CellProfiler and the Icy software to achieve further statistical analysis of the segmented cells. Plantseg, Omnipose, and Icy showed limitations in working with specific types of cells. In addition, the adaptability of different software to preprocessing operations varies considerably, and it is not yet possible to choose a uniform preprocessing method to evaluate the performance of software under different datasets. Therefore, the specific requirements of the dataset and application scenarios should be considered when selecting the right software.

This study highlights the need for continuous development and improvement of cell-image-segmentation software. As technology continues to advance, further enhancements in algorithmic methods and optimization techniques may improve the performance of different datasets. In the future, it is expected that general cell-segmentation software with good segmentation results and advanced functions will be developed. These software tools can be used interactively with other software, such as the CellProfiler platform using Cellpose, StarDist, and other DL models for cell segmentation through plug-ins. The segmentation output generated by Icy can be imported into ImageJ for further statistical analysis and processing. It can also be controlled as a process script, which can analyze the cell structure more accurately and effectively and better serve the related biological research.

## Figures and Tables

**Table 1:** Basic information of the evaluated software tools.

Tool	First Release Time	Version	Programming Language	Dependent Library	Architecture	Pre-Trained Model	GUI	Plugins for Other Tools	Function	Paper	Average Yearly Citations *
CellProfiler	2006	4.2.6	Python	Scikit-image	NA	NA	1	NA	Cell count, size, protein levels cell or organelle shape, and subcellular patterns of DNA or protein staining	[17]	311.6
Icy	2011	2.4.3.0	Java	Vtk, TensorFlow	NA	NA	1	NA	Visualize, annotate, and quantify bioimaging data	[14]	121.2
StarDist	2018	0.8.5	Python	TensorFlow	U-net	2D_paper_DSB2018	0	ImageJ/Fiji, Icy, KNIME, QuPath, Napari	Object detection, multi-class prediction	[19,20]	165.2
DeepCell	2018	0.12.9	Python	TensorFlow	DCNN	NuclearSegmentation	0	ImageJ/Fiji	Cell division, counting, classification, tracking, natural language processing, speech recognition	[23,24,25,26]	75.3
Cellpose	2020	2.2.3	Python	Pytorch	U-net	cyto	1	CellProfiler, Napari	Cell and nucleus segmentation	[21,22]	432
Omnipose	2022	1.0.6	Python	Pytorch	U-net	cyto2_omni	1	Napari	Bacterial cell segmentation	[27]	79.0
Plantseg	2020	1.6.0	Python	Pytorch	U-net	confocal_PNAS_2d	1	Napari, Ilastik	Cell boundary predictions, graph partitioning	[28]	51.3
Ilastik	2011	1.4.0.post1	Python	Scikit-image	NA	NA	1	ImageJ/Fiji	Pixel classification, autocontext, object classification, carving, multicut, counting, tracking	[29,30]	110.1

^*^ The analysis was performed on 5 December 2023 using Google Scholar. NA stands for not available or too diverse to be described individually.

**Table 2:** The user-friendliness of the software. Group A: documentation. Group B: usability. Group C: segmentation mode. Group D: output.

Group		CellProfiler	Icy	StarDist	DeepCell	Cellpose	Omnipose	Plantseg	Ilastik
A	User guide/handbook	✓	✓	✓	✓	✓	✓		✓
	website	✓	✓		✓	✓	✓		✓
	Video tutorial	✓	✓	✓	✓	✓			✓
	Community support	✓	✓	✓	✓	✓	✓	✓	✓
	Test dataset/demo	✓	✓	✓	✓	✓	✓	✓	✓
	Open-source	✓	✓	✓	✓	✓	✓	✓	✓
B	No programming experience is required	✓	✓	✓	✓	✓	✓	✓	✓
	Intuitive visualization settings	✓	✓		✓	✓	✓	✓	✓
	Portability on Win/Linux/Mac	✓	✓	✓	✓	✓	✓	✓	✓
C	Manual	✓	✓		✓	✓	✓		✓
	Interactive	✓	✓	✓	✓	✓	✓	✓	✓
	Automated	✓	✓	✓	✓	✓	✓	✓	
D	2D rendering	✓	✓	✓	✓	✓	✓		✓
	2D binary mask	✓	✓	✓	✓	✓	✓	✓	✓
	Feature statistics	✓	✓			✓	✓		✓

**Table 3:** The details of the three publicly available datasets.

Dataset	Description	Train	Test	RAM (MB)	Channels (n)	Url
DSB2018	one challenging dataset of diverse fluorescence microscopy images	447	50	384.48	1	https://www.kaggle.com/competitions/data-science-bowl-2018/data (accessed on 8 February 2024)
Cellpose_cyto	one dataset consists of fluorescent cytoplasmic markers, confocal imaging, brightfield microscopy, and non-microscopy images	540	68	161.30	3	https://www.cellpose.org/dataset (accessed on 8 February 2024)
PhC-C2DL-PSC	pancreatic stem cells on a polystyrene substrate	600	600	230	1	http://celltrackingchallenge.net/2d-datasets (accessed on 8 February 2024)

**Table 4:** Quantitative comparison of the performance of open-source software tools selected for the study on DSB2018 datasets.

	Software	N_true	N_pred	Precision	Recall	Accuracy	F1	F1-Based Rank
No Preprocessing	Cellpose	49	42	0.9387	0.8227	0.7948	0.8725	2
	Omnipose	49	29	0.7825	0.4421	0.4058	0.5387	3
	StarDist	49	49	0.9314	0.9278	0.8723	0.9283	1
	Plantseg	49	3	0.0050	0.0014	0.0011	0.0022	5
	Icy	49	35	0.5224	0.4302	0.3317	0.4640	4
Preprocessing	Cellpose	49	48	0.9197	0.8959	0.8408	0.9066	1
	Omnipose	49	29	0.7608	0.5150	0.4919	0.5945	5
	StarDist	49	50	0.8727	0.9117	0.8177	0.8894	2
	DeepCell	49	49	0.7641	0.8025	0.6625	0.7802	4
	Plantseg	49	3	0.0007	0.0003	0.0002	0.0005	7
	Icy	49	38	0.5300	0.4937	0.3770	0.5042	6
	CellProfiler	49	47	0.8087	0.8215	0.7058	0.8089	3

**Table 5:** Quantitative comparison of the performance of selected open-source software tools on the Cellpose_cyto datasets.

	Software	N_true	N_pred	Precision	Recall	Accuracy	F1	F1-Based Rank
No Preprocessing	Cellpose	106	65	0.8596	0.6456	0.6102	0.7138	1
	Omnipose	106	87	0.7666	0.6579	0.5593	0.6941	2
	StarDist	106	116	0.5299	0.5609	0.3844	0.5142	3
	Plantseg	106	90	0.1421	0.1855	0.1059	0.1534	5
	Icy	106	63	0.1973	0.1427	0.1034	0.1584	4
	Ilastik	106	60	0.0420	0.0474	0.0230	0.0370	6
Preprocessing	Cellpose	106	89	0.7552	0.6637	0.5798	0.6929	1
	Omnipose	106	5	0.1800	0.0448	0.0384	0.0639	7
	StarDist	106	108	0.3037	0.3575	0.2035	0.3072	3
	DeepCell	106	129	0.3550	0.4596	0.2674	0.3847	2
	Plantseg	106	110	0.1175	0.1476	0.0830	0.1251	6
	Icy	106	87	0.2103	0.1831	0.1260	0.1903	5
	CellProfiler	106	186	0.1776	0.2443	0.1267	0.1914	4

**Table 6:** Quantitative performance comparison of two software tools (Cellpose and StarDist) on 2D datasets.

Dataset	Software	N_true	N_pred	Precision	Recall	Accuracy	F1
DSB2018	StarDist	49	51	0.5707	0.6183	0.4566	0.5912
	Cellpose (untrained)	49	47	0.5900	0.5647	0.4623	0.5763
	Cellpose (trained)	49	45	0.7316	0.6791	0.5776	0.7026
Cellpose_cyto	Cellpose	106	89	0.7552	0.6637	0.5798	0.6929
	StarDist (untrained)	106	108	0.3037	0.3575	0.2035	0.3072
	StarDist (trained)	106	93	0.7050	0.6708	0.5426	0.6739

**Table 7:** Quantitative comparison of the performance of selected open-source software tools on the PhC-C2DL-PSC datasets.

	Software	N_true	N_pred	Precision	Recall	Accuracy	F1	F1-Based Rank
No Preprocessing	Cellpose	237	118	0.5919	0.4410	0.3504	0.4857	2
	StarDist	237	241	0.5025	0.5935	0.3668	0.5353	1
	DeepCell	237	112	0.0887	0.0503	0.0330	0.0634	5
	Icy	237	142	0.5427	0.3894	0.2992	0.4481	3
	CellProfiler	237	349	0.2179	0.3580	0.1537	0.2643	4
Preprocessing	Cellpose	237	75	0.4069	0.2312	0.1832	0.2847	3
	StarDist	237	175	0.5787	0.5327	0.3728	0.5383	1
	DeepCell	237	61	0.0596	0.0206	0.0154	0.0301	5
	Icy	237	103	0.4279	0.2607	0.2015	0.3171	2
	CellProfiler	237	242	0.2238	0.2804	0.1386	0.2424	4

**Table 8:** Quantitative comparison of computer resource consumption of the selected cell-segmentation software.

Software	on GPU or CPU	DSB2018		Cellpose_cyto	
		Time (s)	Memory (MB)	Time (s)	Memory (MB)
Cellpose	GPU	32.89	5383.89	48.85	5423.26
Omnipose	GPU	57.87	6320.05	45.17	6315.86
StarDist	GPU	85.43	4533.08	178.99	4628.75
DeepCell	GPU	110.43	6922.44	112.24	7254.32
Plantseg	GPU	293.6	4234.08	375.17	4274.43
Icy	CPU	132.85	840.15	221.01	851.24
CellProfiler	CPU	660.02	625.86	900.34	699.18

## Data Availability

The data are contained within the article.

## Abbreviations

The following abbreviations are used in this manuscript:

ML	Machine Learning
DL	Deep Learning
GUI	Graphical User Interface
CNN	Convolutional Neural Network
DCNN	Deep Convolutional Neural Network
